# A novel proteasome inhibitor NPI-0052 as an anticancer therapy

**DOI:** 10.1038/sj.bjc.6603406

**Published:** 2006-10-17

**Authors:** D Chauhan, T Hideshima, K C Anderson

**Affiliations:** 1 Department of Medical Oncology, Harvard Medical School, Dana Farber Cancer Institute, The Jerome Lipper Multiple Myeloma Center, Boston, MA 02115, USA

**Keywords:** protein degradation, proteasomes, multiple myeloma, novel therapy, apoptosis, drug resistance

## Abstract

Proteasome inhibitor Bortezomib/Velcade has emerged as an effective anticancer therapy for the treatment of relapsed and/or refractory multiple myeloma (MM), but prolonged treatment can be associated with toxicity and development of drug resistance. In this review, we discuss the recent discovery of a novel proteasome inhibitor, NPI-0052, that is distinct from Bortezomib in its chemical structure, mechanisms of action, and effects on proteasomal activities; most importantly, it overcomes resistance to conventional and Bortezomib therapies. *In vivo* studies using human MM xenografts shows that NPI-0052 is well tolerated, prolongs survival, and reduces tumour recurrence. These preclinical studies provided the basis for Phase-I clinical trial of NPI-0052 in relapsed/refractory MM patients.

The systemic regulation of protein synthesis and protein degradation is essential for normal cellular functioning. The ubiquitin–proteasome pathway mediates intracellular protein degradation: first, protein is marked with a chain of small polypeptides called ubiquitin; E1 ubiquitin enzyme then activates ubiquitin and links it to the ubiquitin-conjugating enzyme E2 in an ATP-dependent manner; E3 ubiquitin ligase then links the ubiquitin molecule to the protein; a long polypeptide chain of ubiquitin moieties is formed; and finally, a multi-enzyme proteolytic complex 26S proteasome degrades protein into small fragments in an ATP-dependent manner. (Ciechanover and Schwartz, 1998; Goldberg and Rock, 2002; Pickart, 2004). The 26S proteasome contains a proteolytic core 20S and 19S regulatory subunits (Elliott and Ross, 2001; Goldberg and Rock, 2002; Ciechanover, 2003). The structure of the 20S core consists of four stacked rings: two central beta rings that are surrounded by two alpha rings, each consisting of seven subunits (Figure 1). The 19S regulatory complex, consisting of a lid and a base, binds to form the 20S proteasome holoenzyme. The lid region of 19S recognises ubiquitinated proteins, whereas the base, which caps the end of the 20S core contains six ATPase that regulates unfolding of the protein substrates and directing them into the catalytic chamber of the 20S core. The 20S proteasome triggers the protein breakdown via its proteolytic activities. Mutagenesis and structural studies show that beta-subunits of the 20S proteasome have amino-terminal threonines that serve as catalytic active sites (nucleophiles) (Figure 1). The three major proteolytic activities are: *β*-5, chymotrypsin-like (CT-L) that cleaves after hydrophobic residues; *β*-2, trypsin-like (T-L) that cleaves after basic residues; and *β*-1, caspase-like (C-L), which cleaves after acidic residues (Schechter and Berger, 1967; Cardozo, 1993; Lupas *et al*, 1993; Orlowski *et al*, 1993). In addition, these three constitutive proteasome activities have corresponding inducible immunoproteasomes *β*-5i, *β*-1i, *β*-2i with different specificity (Uebel and Tampe, 1999; Kessler *et al*, 2001).

The substrates of the 26S proteasome include proteins mediating various cellular functions such as transcription, stress response, cell cycle regulation, oncogenesis, ribosome biogenesis, cellular differentiation, and DNA repair (Ciechanover and Schwartz, 1998). Defects in the complex biochemical machinery of UBP signalling pathway are linked to the pathogenesis of many human diseases (Myung *et al*, 2001). Targeting UBP signalling pathway has therapeutic implication. For example, blockade of proteasome activity results in stabilisation of inhibitory proteins, thereby abrogating growth/survival and triggering apoptosis. Our preclinical and clinical studies in multiple myeloma (MM) validated 20S as a therapeutic target and led to the FDA approval of the first proteasome inhibitor Bortezomib (Velcade) for treatment of relapsed/refractory MM (Hideshima and Anderson, 2002; Chauhan *et al*, 2005b). Bortezomib therapy showed a remarkable overall response rate (35%) in MM patients; however, it can be associated with toxicity and development of drug resistance. Nonetheless, the success of Bortezomib as an anticancer therapy renewed the interest in discovery and development of other novel proteasome inhibitors. Specifically, delineation of mechanisms mediating interaction of proteasome inhibitors with 20S proteasome will allow designing potent and therapeutically efficacious proteasome inhibitors. In this context, our recent study characterised a novel proteasome inhibitor NPI-0052, a small molecule derived from fermentation of Salinospora, a new marine Gram-positive actinomycete (Feling *et al*, 2003; Macherla *et al*, 2005; Chauhan *et al*, 2005a). Importantly, NPI-0052 is distinct from Bortezomib, orally bioactive, and an equipotent antitumour agent (Chauhan *et al*, 2005a).

## CHEMICAL STRUCTURE AND PROTEASOME ACTIVITY PROFILE OF NPI-0052 VIS-À-VIS OTHER PROTEASOME INHIBITORS

The proteasome inhibitors can be categorised into two groups: natural products or synthetic analogues. The synthetic inhibitors are peptide-based agents with varied pharmacophores and have been classified as peptide aldehydes, peptide boronates, peptide benzamides, peptide vinyl sulphones, and peptide alpha-ketoaldehydes and peptide-alpha-ketoamides (Adams *et al*, 1999; Adams, 2004; Chauhan *et al*, 2005b). A well-characterised example of this category of peptide-based proteasome inhibitor is Bortezomib, a boronic acid dipeptide derivative which inhibits proteasome function in a reversible manner via interaction of boronic acid at the C-terminal of Bortezomib with an active threonine site in the proteasome (Adams *et al*, 1999). On the other hand, the natural product proteasome inhibitors exhibit scaffolds of core structures and pharmacophores. Examples of this category include linear peptide epoxyketones, peptide macrocycles, gamma-lactam thiol ester, and fungal epipolythioldioxopiperazine toxin.

**Figure 1:**
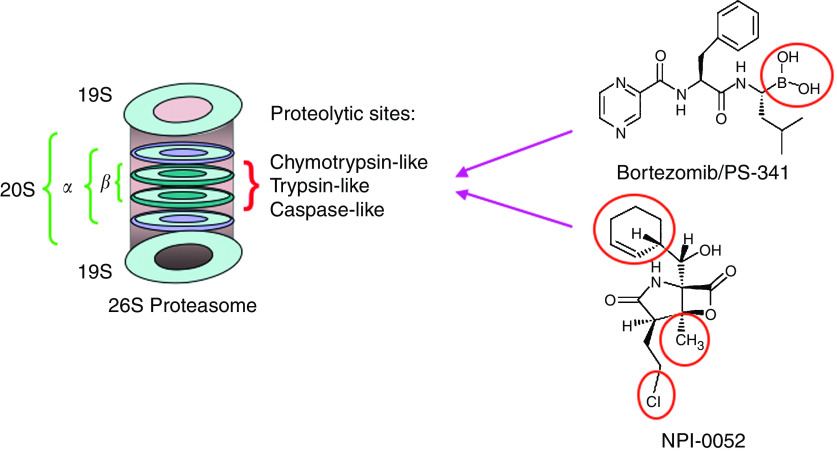
Schematic representation of 26S proteasome composition: The three primary proteolytic activities (CT-L, T-L, and C-L) reside within the *β*-subunits of 20S and interact with proteasome inhibitors. Bortezomib and NPI-0052 are structurally distinct with different active sites.

NPI-0052 is a nonpeptide proteasome inhibitor with structural similarity to Omuralide (Corey and Li, 1999), a beta-lactone derived from naturally occurring lactacystin. However, in contrast to Omuralide, NPI-0052 has a uniquely methylated C3 ring juncture, chlorinated alkyl group at C2, and cyclohexene ring at C5 (Figure 1). Structure–activity relationship studies showed that (1) replacement of chloroethyl group with unhalogenated substituents resulted in loss of potency in cell-based assays, and (2) halogen exchange and cyclohexene ring epoxidation were well tolerated, although some stereochemical modifications markedly attenuated activity (Macherla *et al*, 2005). These data suggested that whereas the beta-lactone is a key pharmachophre of both Omuralide and NPI-0052, the enhanced potency of NPI-0052 is due to the substituents in bicyclic ring system. A recent report describing the crystal structure of NPI-0052 in complex with the 20S proteasome revealed an important consequence of beta-lactone ring opening and a mechanism of irreversible binding vis-à-vis omuralide (Groll *et al*, 2006). Collectively, these structural differences account for the enhanced *in vitro* and *in vivo* potency of NPI-0052 compared to Omuralide (Feling *et al*, 2003; Macherla *et al*, 2005; Groll *et al*, 2006).

NPI-0052 inhibits proteasome activity by covalently modifying the active site threonine residues of the 20S proteasome (Corey and Li, 1999). Comparative study of the effect of NPI-0052 and Bortezomib on proteasomal activities using purified human erythrocyte 20S proteasomes and flurogenic substrates shows that (1) NPI-0052 inhibits CT-L and T-L activities at much lower concentrations than Bortezomib, and (2) higher concentrations of NPI-0052 than Bortezomib are required to inhibit C-L activity (Chauhan *et al*, 2005a). Thus, Bortezomib and NPI-0052 differentially affect all three proteasome activities: NPI-0052 at lower concentrations targets CT-L and T-L, whereas Bortezomib predominantly affects CT-L and C-L. *In vivo* animal studies using whole-blood lysates showed that NPI-0052 completely blocked CT-L activity, which was recoverable by day 7; whereas inhibition of CT-L activity is restored at 24 h after Bortezomib. A 50% inhibition of T-L activity is noted upon NPI-0052 exposure, which is restored by day 7; whereas Bortezomib increases T-L activity, which remains elevated even at day 7. In the context of C-L activity, the profiles of both NPI-0052 and Bortezomib showed similarity, that is, marked inhibition upon initial exposure and recovery at day 7. The likely reason for the late recovery of proteasome activity after NPI-0052 may be owing to its binding characteristic to the 20S proteasome. Nevertheless, the ability of NPI-0052 to trigger sustained inhibition of CT-L, T-L and C-L (up to 7 days) has clinical implications, that is, less frequent administration in patients. Of note, Bortezomib is currently given twice weekly to patients to achieve maximal CT-L inhibition (75–78%). The CT-L activity inhibition in peripheral blood cells of patients is noted within an hour of Bortezomib administration and recoverable before the next dose (Adams, 2002; Hamilton *et al*, 2005).

As noted above, both NPI-0052 and Bortezomib differentially inhibit proteasome activities. Whether inhibition of two or all three proteolytic activities is therapeutically advantageous is now being defined. A recent report examined the role of proteolytic sites using site specific inhibitors on the degradation of several model unfolded proteins (histones, casein, aged calmodulin, and denatured ovalbumin) by purified mammalian 26S proteasome (Kisselev *et al*, 2006). This study demonstrates that therapeutic concentrations of Bortezomib which inhibit 75% of CT-L activity only result in partial protein breakdown, that is 10–25%, suggesting that protein breakdown machinery remains functional despite Bortezomib exposure. Furthermore, a simultaneous inhibition of multiple proteasome activities is a prerequisite for a significant (i.e., >50%) proteolysis (Kisselev *et al*, 2006). Another study showed that a 50% inhibition of cystic fibrosis transmembrane conductance regulator degradation in reticulocytes extracts required concurrent blockade of CT-L and C-L proteasome activites (Oberdorf *et al*, 2001).

*In vitro* studies using purified 20S proteasomes showed that NPI-0052 has lower EC_50_ for T-L than does Bortezomib. *In vivo* animal model studies show marked inhibition of T-L activity in response to NPI-0052, whereas Bortezomib enhances T-L proteasome activity. A further confirmation of the ability of NPI-0052 to block proteasomal activity was derived from experiments using a novel methodology (instead of conventional use of flourogenic substrate) to assess proteasome activity in MM cells. Specifically, competition experiments between NPI-0052 and a cell-permeable proteasome inhibitor dansylAhx_3_L_3_VS that covalently modifies all active proteasome subunits showed that NPI-0052 at the IC_50_ doses for MM cells reduced the dansylAhx_3_L_3_VS-labelling of the *β*-5 (represents CT-L activity), *β*-1 (represents C-L activity), and *β*-2 (represents T-L activity) subunits (Berkers *et al*, 2005). Higher doses of Bortezomib are required to inhibit CT-L and C-L activity, whereas little, if any, inhibition of T-L activity was noted. Importantly, our study showed that MM cells exhibit higher constitutive levels of T-L proteasome activity than either CT-L or C-L activities (Crawford *et al*, 2006). These data, coupled with the results that NPI-0052, but not Bortezomib, efficiently inhibits CT-L+T-L activities (Chauhan *et al*, 2005a), suggest that NPI-0052 may trigger more proteolysis than Bortezomib in MM cells.

Besides the constitutive proteasome activities, NPI-0052 and Bortezomib also affect the immunoproteasomes. Alterations in immunoproteasome may have clinical implications by modulating the immune response in MM patients. Our data suggest that both NPI-0052 and Bortezomib differentially affect constitutive and immunoproteasome in MM.1S MM cell line (Chauhan *et al*, 2005a). A novel methodology was applied to measure proteasome activity by Western blot analysis using dansyl-Ahx_3_L_3_VS as a probe (Berkers *et al*, 2005). NPI-0052 inhibits *β*-5i, *β*-2i, and *β*-1i activities in MM cells. Slightly higher concentrations of Bortezomib were required to achieve inhibition of *β*-5i, *β*-2i, and *β*-1i. A recent study also showed that Bortezomib inhibits the immunoproteasome in MM cells (Altun *et al*, 2005). It is likely that blockade of one or more of these proteasomal activities along with the kinetics of inhibition confer therapeutic advantage. Moreover, previous reports showed that different organs/cell types express different ratios of constitutive-, immuno-, and hybrid-proteasomes (Stohwasser *et al*, 1997; Dahlmann *et al*, 2000; Crawford *et al*, 2006). Our ongoing studies using MM patient cells and a panel of MM cell lines are addressing these questions by designing more sensitive and specific proteasome probes against each proteasome subunits. These studies will further delineate the differential impact of NPI-0052 and Bortezomib on both constitutive proteasome activities and immunoproteasomes.

## *IN VITRO* AND *IN VIVO* ANTITUMOUR ACTIVITY OF NPI-0052

Initial screening of NPI-0052 against the NCI panel of 60 tumour cell lines showed GI_50_ of <10 nM in all cases (Feling *et al*, 2003). In concert with these observations, our data showed that (1) NPI-0052 induces apoptosis in MM cells sensitive and resistant to conventional and Bortezomib therapies; and (2) IC50 of NPI-0052 for MM cells is within the low nanomolar concentration (Chauhan *et al*, 2005a). Importantly, NPI-0052 similarly triggered apoptosis in purified tumour cells from several MM patients relapsing after various prior therapies including Bortezomib and thalidomide. Although the cells from Bortezomib-refractory patients remain sensitive (15–50%) to the treatment with Bortezomib *in vitro*, our data suggest that NPI-0052 is a more potent inducer of these cells. The variable *in vitro* cytotoxicity of NPI-0052 and Bortezomib may be owing to their distinct mode of action in MM cells including differential effects on proteasome activities. Given that therapeutic concentrations of Bortezomib primarily target CT-L activity, the other two proteolytic activities may compensate, thereby maintaining the functionality of proteasome. On the other hand, NPI-0052 inhibits all three proteolytic activities, thereby achieving maximal proteolysis. Moreover, mechanisms conferring Bortezomib resistance may not be effective against NPI-0052. Nonetheless, our study suggests that NPI-0052 is a potent inducer of MM cells apoptosis in tumour cells obtained from Bortezomib-refractory MM patients. Development of Bortezomib-resistant MM cell lines will further elucidate this issue.

Drug resistance in MM cells is conferred, in part, by the bone marrow (BM) microenvironment. Adhesion of MM cells with BM stromal cells (BMSCs) induces cytokine secretion, which induce paracrine growth of MM cells and protect against drug-induced apoptosis (Hideshima and Anderson, 2002). Our findings show that NPI-0052, like Bortezomib, inhibits the secretion of BMSCs-derived MM cell growth factor IL-6 within the BM milieu, without affecting the viability of MM BMSCs. Furthermore, NPI-0052 triggers MM cell apoptosis even in the presence of IL-6 or IGF-I. Similarly, NPI-0052 abrogates VEGF-triggered migration (Podar *et al*, 2002) of MM cells, suggesting NPI-0052 as an antimigratory agent. Whether MM cell interaction with BM components affects the proteasome activity profiles of either MM cells or BMSCs is unclear. Of note, we have shown that MM-BMSC binding upregulate transcript levels of 26S proteasome subunits, which may serve to meet the increased demand for protein synthesis and protein degradation in MM cells. This may also explain higher sensitivity of MM cells to proteasome inhibitor exposure in the BM microenvironment.

Given that protein degradation is required for normal cellular progression, it is imperative to examine the effect of proteasome inhibitors on normal cell viability and proteasome activity. Comparative examination of the effects of NPI-0052 and Bortezomib on normal lymphocytes showed that NPI-0052 does not significantly decrease normal lymphocyte viability at the IC_50_ doses for MM cells, with modest effects only at higher concentrations of NPI-0052. By contrast, Bortezomib decreased the survival of lymphocytes at the concentrations close to the IC_50_ doses for MM cells. The impact of NPI-0052 on proteasome activity showed that NPI-0052 inhibits CT-L activity, even at the doses that does not trigger apoptosis in MM cells. Earlier observations that Bortezomib inhibits 20S proteasome activity in murine WBCs at 1 h post injections, and that a similar degree of proteasome inhibition was noted in blood from responders *vs* non-responders to Bortezomib therapy (Adams, 2002; Richardson, 2004), suggest that inhibition of proteasome activity in blood may not correlate to cytotoxicity. The mechanism whereby proteasome inhibitors block proteasome activity without triggering apoptosis is unclear. One possibility is that proteasome inhibition in normal cells is compensated by alternative intact protein breakdown mechanisms such as lysosomal pathways, which will allow for uninterrupted protein degradation. In addition, cancer cells, in contrast to normal cells, have abnormal protein synthesis (e.g., high immunoglobulins secretion in MM), resulting in increased dependence on proteasomes to degrade misfolded proteins (which also explains the increased sensitivity of MM cells to proteasome inhibition). Nonetheless, the above data suggest that NPI-0052, in contrast to Bortezomib, is likely to have less toxic effects than Bortezomib on normal lymphocytes cells.

Examination of the *in vivo* efficacy of NPI-0052 using a human plasmacytoma xenograft mouse model showed potent antitumour activity of NPI-0052 given orally (LeBlanc *et al*, 2002; Chauhan *et al*, 2005a). Treatment of tumour bearing mice with NPI-0052, but not vehicle alone, inhibited MM tumour growth and prolongs survival of these mice. NPI-0052 doses were well tolerated by mice, without significant weight loss or any neurological behavioural changes. Importantly, analysis at day 300 showed no recurrence of tumour in 57% of the NPI-0052-treated mice. A head-to-head examination of NPI-0052 and Bortezomib showed that both agents reduced tumour progression and prolonged survival.

## MECHANISTIC INSIGHTS INTO NPI-0052 TRIGGERED CELL DEATH

Nuclear factor-kappa B (NF-*κ*B) is a major growth and survival signalling pathway in MM; indeed, the initial rationale for therapeutic use of Bortezomib was, in part, based on its ability to inhibit NF-*κ*B activation (Russo *et al*, 2001; Adams, 2002; Hideshima *et al*, 2002). Comparative analysis of the effect of NPI-0052 and Bortezomib on NF-*κ*B showed that NPI-0052 is a more potent inhibitor of NF-*κ*B and related cytokine transcription and secretion than Bortezomib. These findings show that NPI-0052, like Bortezomib, targets NF-*κ*B. Importantly, blockade of NF-*κ*B also have implication in modulating immune responses, as NF-*κ*B play a role during T-cell activation and function. A recent study showed that blockade of NF-*κ*B using Bortezomib triggered cell death in activated T cells along with decreased production of Th1 cytokines (INF-*γ* and IL-2) (Blanco *et al*, 2006). Because NPI-0052 is an irreversible inhibitor, it may similarly inhibit T-cell function via NF-*κ*B modulation; and, our current studies are examining this question using MM patient-derived T-cells and normal resting or activated T cells.

**Figure 2:**
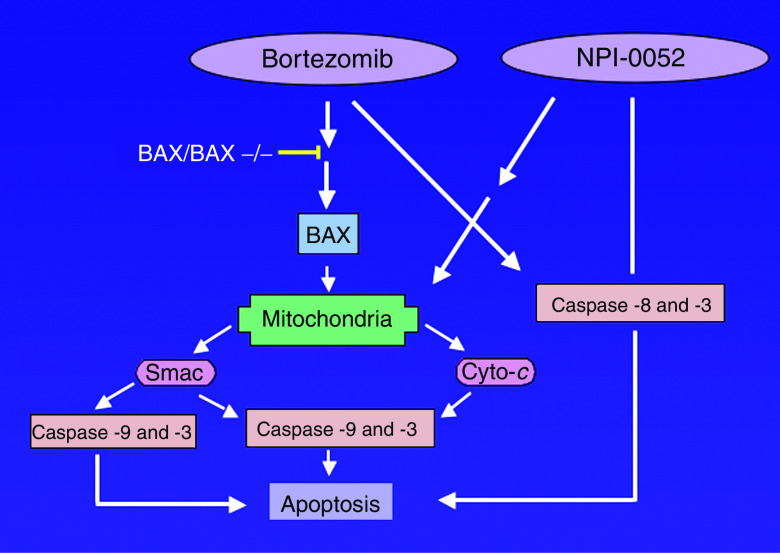
NPI-0052- and Bortezomib-induced cell-death signalling pathways. Both NPI-0052 and Bortezomib trigger intrinsic (caspase-9-mediated) and extrinsic (caspase-8-mediated) apoptotic signalling pathways. Deletion of Bax/Bak results in significant loss of response to Bortezomib, but not NPI-0052.

Our studies in MM cells showed that NPI-0052-, like Bortezomib, -induced apoptosis, is associated with activation of both extrinsic (caspase-8-mediated) and intrinsic (caspase-9-mediated) cell-death signalling pathways (Chauhan *et al*, 2005a). Specifically, NPI-0052-induced MM cell death correlates with (1) decrease in ΔΨ_m_; (2) increase in O2− production; (3) the release of cytochrome *c* and smac from mitochondria to cytosol; and (4) activation of caspase-9, caspase-8, and caspase-3, followed by PARP cleavage. The requirement for caspase-8 *vs* caspase-9 during NPI-0052- and Bortezomib-induced apoptosis was further defined using both biochemical inhibitors and dominant-negative strategies. Our data demonstrated that (1) NPI-0052-induced MM cell apoptosis is predominantly mediated by caspase-8; and (2) Bortezomib-induced apoptosis requires both caspase-8 and caspase-9 activation (Figure 2). These findings suggest that NPI-0052 relies more on FADD–caspase-8 signalling axis than does Bortezomib, further confirming differential action of NPI-0052 *vs* Bortezomib in MM cells. Studies using Bax/Bak DKO further showed that, in contrast to NPI-0052-, Bortezomib-mediated cell death requires mitochondrial apoptotic signalling via Bax and Bak. Moreover, ectopic expression of antiapoptotic protein Bcl-2 provided more protection against Bortezomib than NPI-0052 in MM cells.

The mechanistic differences between NPI-0052 and Bortezomib, that is, their effect on proteasome activities and their dependence on specific apoptotic signal transduction pathways, provides the basis for combination regimens for the treatment of MM. As observed in our study (Chauhan *et al*, 2005a), the combination of NPI-0052 with Bortezomib induced synergistic anti-MM activity, without significantly affecting the viability of normal lymphocytes. The mechanisms mediating enhanced cytotoxicity of the combination regimen may simply reflect higher levels of proteasome inhibition with the two-drug regimen and/or differential apoptotic signalling pathways. Nonetheless, the benefit of combined regimen can only be established after a future clinical trial.

## CONCLUDING REMARKS

As the ubiquitin–proteasome pathway regulates many essential cellular processes, proteasome inhibitors offer a great promise as therapeutic agents. Our study has evaluated the potential of a novel proteasome inhibitor NPI-0052 as an anticancer agent. NPI-0052 inhibits proteasome activity both *in vitro* and *in vivo* at clinically achievable doses, and induces a different proteasome inhibition profile than Bortezomib. NPI-0052 is a more potent inhibitor of NF-*κ*B and related cytokine secretion than Bortezomib. NPI-0052 induces apoptosis in MM cells resistant to conventional and Bortezomib therapies, without significantly affecting normal lymphocyte viability. Importantly, NPI-0052, like Bortezomib, kills MM cells even in the presence of protective BM microenvironment without affecting the viability of BM cells. Moreover, NPI-0052 overcomes drug resistance conferred by the antiapoptotic protein Bcl-2. NPI-0052 given orally inhibits MM tumour growth *in vivo* as well as prolongs survival, without recurrence of tumour in 57% mice. Biochemical and genetic evidence indicates that NPI-0052, in contrast to Bortezomib, relies more on FADD-caspase-8-mediated cell death signalling. Finally, combinations of low doses of NPI-0052 and Bortezomib trigger synergistic anti-MM activity. Overall, our study provides the rationale for clinical protocols evaluating NPI-0052 to inhibit tumour cell growth, overcome conventional chemotherapy and Bortezomib resistance, limit toxicity profiles, and improve patient outcome in MM.
